# Soil Aggregate Fungal Network Complexity Drives Soil Multifunctionality During Vegetation Restoration

**DOI:** 10.3390/microorganisms14010161

**Published:** 2026-01-11

**Authors:** Renyuan He, Zhuzhu Luo, Jiahe Liu, Liangliang Li, Lingling Li, Yining Niu, Zhiming Chen, Yaoquan Zhang

**Affiliations:** 1 College of Forestry, Gansu Agricultural University, Lanzhou 730070, China; 18393414923@163.com (R.H.); 13621@gsau.edu.cn (Z.C.); zhangyqgs@163.com (Y.Z.); 2 College of Resources and Environmental Sciences, Gansu Agricultural University, Lanzhou 730070, China; liujiahe@gsau.edu.cn; 3 Grassland Science College, Gansu Agricultural University, Lanzhou 730070, China; 18394797671@163.com; 4 State Key Laboratory of Arid Habitat Crop Science, Lanzhou 730070, China; lill@gsau.edu.cn (L.L.); niuyn@gsau.edu.cn (Y.N.)

**Keywords:** loess plateau, vegetation restoration, soil aggregate, soil multifunctionality, microbial community, microbial network complexity

## Abstract

Vegetation restoration is an effective strategy to improve the ecosystem function of the Loess Plateau. Soil microbiomes play a critical role in maintaining soil multifunctionality (SMF). However, the role of aggregate-scale microbial communities and interactions in regulating SMF during vegetation restoration remains poorly understood. Here, we selected six types of vegetation restoration measures in the Loess Plateau, including natural grassland (NL), *Medicago sativa* (MS), *Hippophae rhamnoides* (HR), *Caragana korshinskii* (CK), *Armeniaca vulgaris* (AV), and *Populus alba* (PA), and used abandoned land (AL) as a control to identify key microbial mechanisms driving SMF at the aggregate scale. The results show that vegetation restoration increased bacterial diversity, fungal network complexity, and SMF, especially in AV. In contrast, fungal diversity and bacterial network complexity exhibited asynchronous dynamics across different-sized aggregates. Soil microbial diversity peaked at micro-aggregates (0.053–0.25 mm), while fungal network complexity increased with decreasing aggregate size. The structural equation model confirmed that fungal community composition in large macro-aggregates (>2 mm) and fungal network complexity in <2 mm aggregates were the key drivers of SMF. Our results emphasize the divergent mechanisms by which microbial properties influence SMF across aggregate sizes, highlighting the importance of fungal communities in maintaining soil ecosystem functions.

## 1. Introduction

The Loess Plateau represents a globally significant ecologically fragile region, characterized by highly erodible loess soils, complex landscapes, sparse vegetation, concentrated summer rainfall, and intensive anthropogenic disturbances. To control soil erosion and enhance regional ecosystem restoration on the Loess Plateau, the Grain-for-Green Program was initiated in the late 20th century, leading to a significant increase in vegetation coverage [1,2]. Vegetation restoration, as one of the processes of land use change, affects soil microbial characteristics by changing plant community composition, net primary productivity, species-specific litter, and root exudates, thereby improving soil ecosystem function (i.e., soil multifunctionality, SMF) [3,4]. Investigating how vegetation restoration influences soil ecological functions has become a central focus in terrestrial ecosystem research.

Soil microorganisms are critical drivers in maintaining ecosystem stability and functionality through their decomposition activities, serving as effective biomarkers for assessing ecosystem restoration [5,6]. Owing to their differential sensitivity to environmental pressures, bacteria and fungi exhibit distinct contributions to SMF. Jin et al. identified soil fungal communities as a key driver of SMF during vegetation restoration in metallic tailings reservoirs [7]. In contrast, Luo et al. found that bacterial diversity exhibited a stronger association with SMF than fungal diversity [8]. This uncertainty in these relationships hinders the identification of microbial predictors for soil multifunctionality. Numerous studies have examined the influence of microbial communities on SMF but have ignored the effects of ecological association between microorganisms on SMF [9,10]. Microbial co-occurrence network analysis assessed the complexity and species interactions within the microbial community [11]. To some extent, using the complexity of microbial networks to predict SMF may be more effective than microbial community composition and diversity [12].

Soil aggregates, the carriers of organic carbon, nitrogen, phosphorus, and other nutrients, are the main sites for material exchange and energy flow. The resulting microscale heterogeneous habitats significantly influence the spatial distribution and ecological functioning of microbial communities [13,14,15]. Previous studies have examined that the compact structure and spatial isolation of micro-aggregates (53–250 μm) limit microbial movement and contact compared to large macro-aggregates (>2 mm) and silt-clay fractions (<53 μm), thereby reducing interspecific competition and thus promoting higher microbial diversity and nutrient use efficiency [16,17]. As we all know that vegetation restoration alters the internal architecture and resource allocation of soil aggregates through organic inputs from litter and fine roots, thereby shaping microbial adaptive strategies and survival mechanisms [18,19]. Therefore, understanding aggregate-scale microbial dynamics and their functional roles during vegetation restoration is critical for designing effective ecological restoration strategies.

To maintain ecosystem stability in the loess hilly gully region, the Grain-for-Green Program was implemented here in the late 20th century, resulting in various vegetation restoration types. These include artificial grasslands (e.g., *Medicago sativa*), artificial shrublands (e.g., *Caragana korshinskii* and *Hippophae rhamnoides*), and artificial forests (e.g., *Armeniaca vulgaris* and *Populus alba*), alongside extensive natural grasslands [20]. Therefore, this study selected abandoned cropland as a control and these six typical vegetation restoration types as research subjects to investigate the microbial driving mechanism of vegetation restoration affecting SMF at the aggregate level. We hypothesized that (1) soil microbial community responses to vegetation restoration varied across aggregate sizes, with diversity highest in micro-aggregates; (2) the complexity of both bacterial and fungal networks collectively drives SMF and varies with soil aggregate size.

## 2. Materials and Methods

### 2.1. Site Description

**Figure 1:**
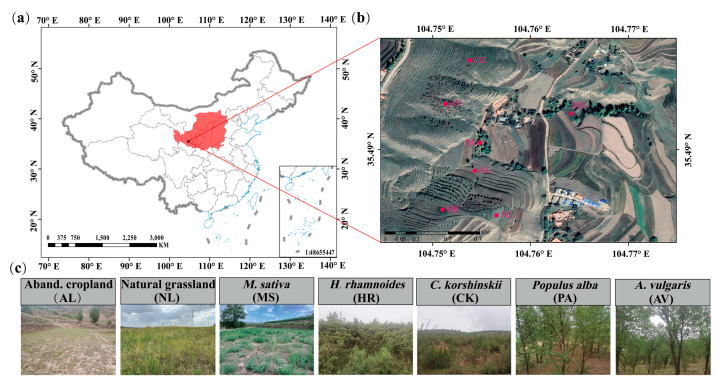
The location of the study area in the Loess Plateau (**a**), the location of the sample plots within the study area (**b**), and the field photographs of the sample plots (**c**).

The study area was located at the Lumacha watershed of Dingxi, Gansu Province (104°74′–104°77′ E, 35°37′–35°50′ N), a secondary tributary of the Zuli River (Figure 1a). This area experiences a temperate continental climate, with a mean annual temperature of 6.4 °C, a mean annual precipitation of 415.2 mm, and a mean annual sunshine of 2426 h. The main type of soil in this area is loess soil developed from loess parent material. The main vegetation types include artificial forests dominated by *Populus alba* and *Armeniaca vulgaris*, artificial shrubbery dominated by *Caragana korshinskii* and *Hippophae rhamnoides*, artificial grassland dominated by *Medicago sativa*, and natural grassland dominated by *Agropyron cristatum* and *Thymus mongolicus* (https://www.plantplus.cn/en, accessed on 5 October 2025) [20].

### 2.2. Experimental Design, Soil Sampling and Preparation

This study selected soil samples from six typical vegetation types as research objects (Figure 1b,c), including natural grassland (NL), *Medicago sativa* (MS), *Hippophae rhamnoides* (HR), *Caragana korshinskii* (CK), *Armeniaca vulgaris* (AV), and *Populus alba* (PA), using abandoned cropland (AL) as a control. Four standard experimental plots of 20 m × 20 m, 5 m × 5 m, and 1 m × 1 m were randomly selected and established in arbor forests, shrublands, and grasslands, respectively. All sample plots are more than 50 m away from each other. The site conditions (including slope, aspect, and altitude) of the standard sample plot are relatively consistent, and a total of 28 sample plots (7 × 4) are set up. The basic situation of the vegetation sample plot is shown in Table S1.

In July 2023, soil samples were collected from 0 to 30 cm depth at each plot using a soil auger following the five-point sampling method. After the removal of gravel, plant residues, and other debris, the soil samples were mixed to form a composite sample.

### 2.3. Soil Aggregate Separation

The fresh soil samples (about 500 g) were air-dried to contain about 15% gravimetric water content, and then the samples were manually broken to <8 mm and divided into two parts. One part was analyzed as bulk soil, and another part was for soil aggregate fractionation using a vibrating screening device (STZS-200, DEDU instruments, Changzhou, China). Briefly, the samples were transferred to a vibrating screening device and sieved at a speed of 500 rpm through sieve sizes (2 mm, 0.25 mm, and 0.053 mm) for 10 min according to [21]. Eventually, soil samples of >2 mm, 0.25–2 mm, 0.053–0.25 mm, and <0.053 mm were used as large macro-aggregates (LM), small macro-aggregates (SM), micro-aggregates (MI), and silt/clay (SC), respectively.

### 2.4. Analysis of Soil Multifunctionality

Fifteen indexes closely related to soil C (organic C, dissolved organic C, easily oxidizable organic C, microbial biomass C, and β-glucosidase), N (total N, nitrate N, ammonium N, microbial biomass N, N-acetylglucosaminidase, and leucine aminopeptidase), and phosphorus (total P, available P, microbial biomass P, and alkaline phosphatase) cycles for quantitative studies. The methods and results of the above indicators are detailed in the Supplementary Material (Table S2, Figure S1). All soil variables were standardized to the range of 0–1 by the following formula [22]:

(1) $$STD=\frac{\left(X-X_{min}\right)}{\left(X_{max}-X_{min}\right)}$$

where *STD* is the standardized value of the variable, *X* is the actual measured value of the target variable, while the *X_min_* and *X_max_* are the minimum and maximum values of the target variable across all samples, respectively. Then the average value of the selected variables was calculated to obtain SMF and C, N, and P cycling functions [23].

### 2.5. DNA Extraction, Sequencing and Data Processing

Microbial DNA was extracted from 0.5 g of fresh soil (140 samples) using the PowerSoil DNA Isolation Kit (MoBio, San Diego, CA, USA), with purity and quality assessed by 1% agarose gel electrophoresis. Subsequently, PCR amplification was conducted in triplicate for both bacterial and fungal communities. The primer sets (338F, 5′-ACTCCTACGGGAGGCAGCAG-3′; 806R, 5′-GGACTACHVGGGTWTCTAAT-3′) were used to amplify the V3–V4 region of the 16S rRNA for subsequent bacterial analysis [24]. The ITS1F (5′-CTTGGTCATTTAGAGGAAGTAA-3′)-ITS2R (5′-GCTGTGTTCTTCATCGATGC-3′) were used to amplify the ITS regions for subsequent fungi analysis [25]. The PCR amplification was conducted under the following conditions: initial denaturation at 95 °C for 5 min; 30 cycles of denaturation at 95 °C for 30 s, annealing at 58 °C for 30 s, and extension at 72 °C for 1 min; followed by a final extension at 72 °C for 5 min. The amplified products were quantified, pooled, and used for library construction. Sequencing was carried out on an Illumina MiSeq PE300 platform (Majorbio, Shanghai, China). Raw paired-end reads were merged, quality-controlled, and filtered before OTU clustering and taxonomic assignment. Microbial diversity indices were then calculated from the resulting OTU table.

### 2.6. Microbial Co-Occurrence Network Analysis

Based on high-throughput sequencing data, we built separate bacterial and fungal co-occurrence networks for all seven vegetation types at the aggregate scale, producing 28 networks for each community type. Prior to network construction, microbial taxa with a relative abundance below 0.01% or present in fewer than three samples were removed. Based on Spearman correlation coefficient ≥0.8 or ≤−0.8 and *p* < 0.05, the network was constructed and visualized by Cytoscape software (v3.4.0, https://cytoscape.org/, accessed on 10 October 2025). The igraph package in R is used to calculate the topological parameters of the co-occurrence network, including nodes, edges, average degree, clustering coefficient, average path length, and graph density [26,27]. Elevated values of key topological metrics (nodes, edges, average degree, clustering coefficient, and graph density), together with a shorter average path length, characterize a more tightly connected and complex microbial network [28]. Therefore, we calculated the mean value of standardized network topological parameters as the network complexity index [29,30]. It is noteworthy that the average path length was calculated as the inverse of the variables before the calculation of the index to align its direction with other complexity parameters.

### 2.7. Statistical Analysis

We performed all statistical analyses for soil biochemical properties, enzyme activities, microbial diversity, and SMF with SPSS version 25.0 (IBM, Armonk, NY, USA). We employed one-way analysis of variance (ANOVA) and least significant difference (LSD) tests to evaluate the effects of vegetation type on soil metrics in both bulk soil and aggregates. A linear mixed-effects model analysis was used to explore the contribution of vegetation types, aggregates, and their interaction due to the change in soil microbial diversity in R (‘glmm.hp’ package) [31]. A partial least squares path model was employed to assess the linkages between soil microbial properties and SMF in R (‘plspm’ package) [32], and its dependability was tested by Goodness of Fit (GoF).

## 3. Results

### 3.1. Soil Biochemical Properties and Soil Multifunctionality

**Figure 2:**
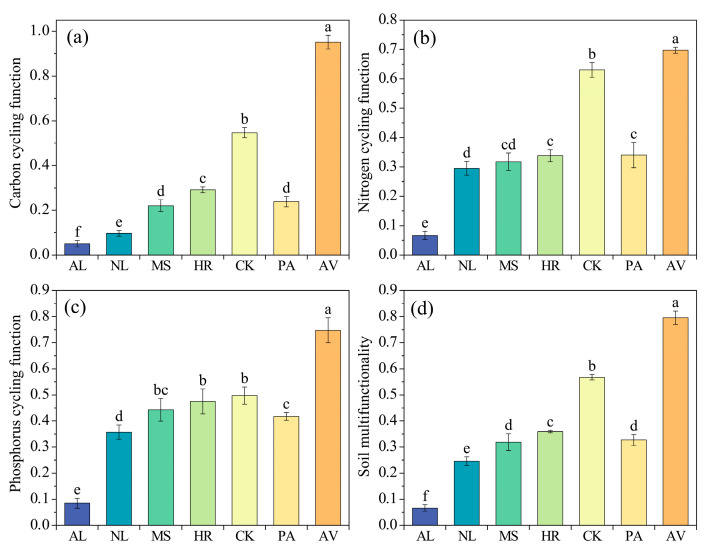
Soil carbon cycling function (**a**), nitrogen cycling function (**b**), phosphorus cycling function (**c**) and multifunctionality (**d**) under different vegetation restoration types. AL: abandoned cropland, NL: natural grassland, MS: *Medicago sativa*, HR: *Hippophae rhamnoides*, CK: *Caragana korshinskii*, PA: *Populus alba*, AV: *Armeniaca vulgaris*. Different lowercase letters indicate significant differences among vegetation types according to the least significant difference (LSD) test (*p* < 0.05).

All vegetation restoration types significantly reduced soil pH, with the lowest value (8.40) observed under AV (Figure S1). However, soil nutrient concentrations, microbial biomass, and extracellular enzyme activities exhibited opposite trends (Figure S1). Compared to AL, the soil C:N ratio decreased by 17.28% and 14.53% in NL and CK, respectively, whereas MS, HR, PA, and AV had no effects (Figure S2, *p* > 0.05). Additionally, all vegetation restoration types significantly increased soil multifunctionality and the C, N, and P cycling functions (Figure 2). Specifically, the soil multifunctionality with NL, MS, HR, CK, PA, and AV was higher than AL by 3.70, 4.79, 5.39, 8.52, 4.91, and 11.94 times, respectively.

### 3.2. Soil Microbial Diversity

**Figure 3:**
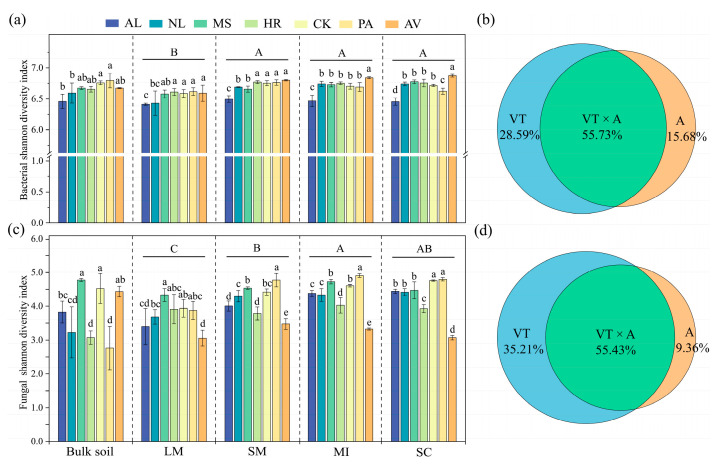
Bacterial and fungal Shannon diversity (**a**,**c**) in bulk soil and aggregates under different vegetation restoration types; the contribution of vegetation types, aggregates, and their interaction to the changes in soil bacterial and fungal diversity (**b**,**d**). AL: abandoned cropland, NL: natural grassland, MS: *Medicago sativa*, HR: *Hippophae rhamnoides*, CK: *Caragana korshinskii*, PA: *Populus alba*, AV: *Armeniaca vulgaris*, LM: large macro-aggregates, SM: small macro-aggregates, MI: micro-aggregates, SC: silt/clay. Different lowercase letters indicate significant differences among vegetation restoration types within the same aggregate size, and different uppercase letters indicate significant differences among aggregate sizes according to the least significant difference (LSD) test (*p* < 0.05).

Vegetation types, aggregate size, and their interactions all had significant effects on the changes in soil microbial α-diversity (Figure 3). Compared to AL, the bacterial Shannon diversity of NL, MS, HR, CK, PA, and AV bulk soil and aggregates increased by 0.29–4.41%, 2.49–4.95%, 3.05–4.65%, 2.72–4.63%, 2.61–5.20%, and 2.80–6.50%, respectively (*p* < 0.05). For fungal Shannon diversity, the bulk soil of MS and CK increased by 24.73% and 18.21%, respectively, whereas HR and PA decreased by 18.21% and 27.84% compared to AL (*p* < 0.05). Similarly, MS and CK enhanced the fungal Shannon index in aggregates, while AV and HR reduced it, with the effect being particularly significant in <2 mm aggregates (*p* < 0.05).

Microbial α-diversity showed differences among aggregates (Figure 3). The bacterial Shannon index was significantly higher in <2 mm aggregates than in large macro-aggregates, whereas the fungal Shannon index was greater in micro-aggregates than in large and small macro-aggregates (*p* < 0.05). Mixed-effects models revealed that vegetation type, aggregate size, and their interaction explained 28.59%, 15.68%, and 55.73% of the variance in bacterial Shannon diversity and 35.21%, 9.36%, and 55.43% of the variance in fungal Shannon diversity, respectively.

**Figure 4:**
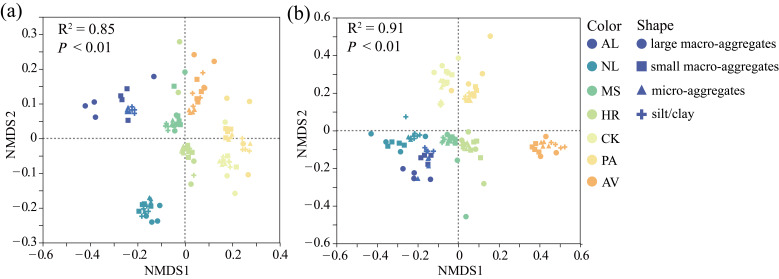
Nonmetric multidimensional scaling (NMDS) ordination of bacterial (**a**) and fungal (**b**) communities in bulk soil and soil aggregates under different vegetation restoration types. AL: abandoned cropland, NL: natural grassland, MS: *Medicago sativa*, HR: *Hippophae rhamnoides*, CK: *Caragana korshinskii*, PA: *Populus alba*, AV: *Armeniaca vulgaris*.

The results of NMDS indicated that both vegetation restoration types and aggregate size significantly influenced microbial β-diversity (Figure 4). Specifically, microbial communities in aggregates of AV and PA were positioned on the positive NMDS1 axis, whereas those of AL, NL, and MS were located on the negative NMDS1 axis.

### 3.3. Microbial Community Compositions

**Figure 5:**
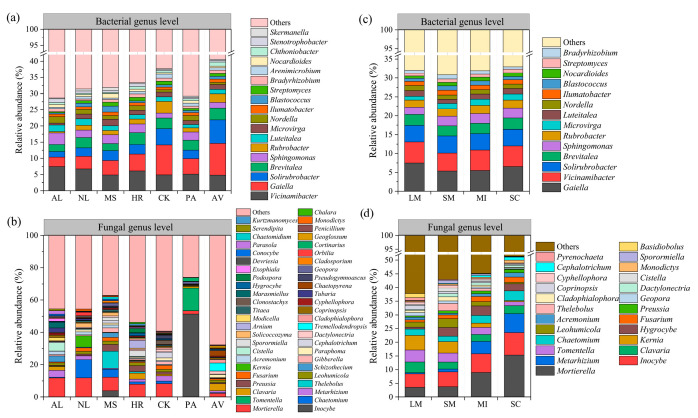
Differences in the composition of bacterial and fungal communities under different vegetation types and aggregate sizes. Bacterial community composition at genus level in the bulk soil under different vegetation types (**a**), bacterial community composition at genus level in different aggregate sizes (**b**), fungal community composition at genus level in the bulk soil under different vegetation types (**c**), fungal community composition at genus level in different aggregate sizes (**d**). AL, NL, MS, HR, CK, PA and AV represent abandoned cropland, grassland, *Medicago sativa*, *Hippophae rhamnoides*, *Caragana korshinskii*, *Populus alba* and *Armeniaca vulgaris*, respectively. LM: large macro-aggregates, SM: small macro-aggregates, MI: micro-aggregates, SC: silt/clay.

High-throughput sequencing revealed bacterial sequences classified into 9 phyla (90 genera) and fungal sequences into 12 phyla (440 genera). At the bacterial genus level, Vicinamibacter predominated in bulk soils of AL (7.51%), NL (6.78%), MS (4.91%), HR (6.15%), and PA (5.16%), whereas Gaiella predominated in CK (9.16%) and AV (9.77%) (Figure 5a). Meanwhile, Gaiella predominated across all aggregates (5.40–7.48%) (Figure 5c). In fungal communities, the relative abundance of Mortierella was the highest in bulk soils of AL (11.73%), NL (11.80%), HR (7.93%), and CK (8.27%), while Thelebolus (10.68%), Inocybe (51.38%), and Tremellodendropsis (4.94%) were found in MS, PA, and AV, respectively (Figure 5b). Among soil aggregates, Kernia predominated in large macro-aggregates and Inocybe in small macro-aggregates, while Mortierella prevailed in both micro-aggregates and silt/clay (Figure 5d).

**Figure 6:**
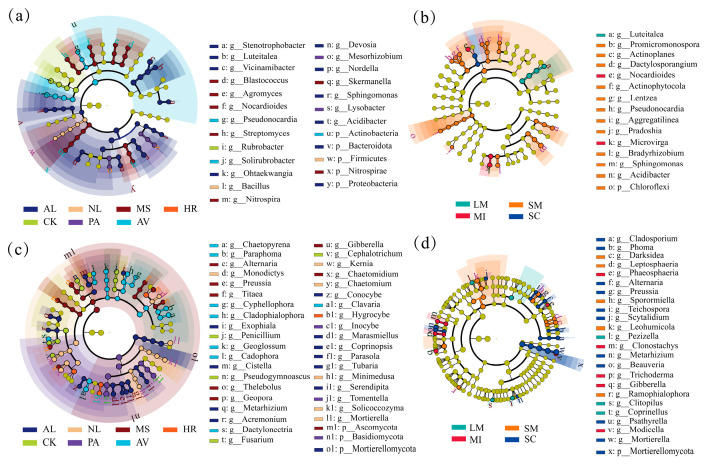
Discrimination of community compositions in soil bacteria (**a**,**b**) and fungi (**c**,**d**) under different vegetation types and aggregate sizes based on linear discriminant analysis effect size (LEfSe). Each small circle at different classification levels represents a classification at that level, and the diameter of the small circle represents the relative abundance. Species with no significant differences are uniformly colored in yellow, while species with significant differences are colored by biomarkers following the group. The species names corresponding to biomarkers are displayed on the right side, with letter numbers corresponding to the figure. AL, NL, MS, HR, CK, PA and AV represent abandoned cropland, grassland, *Medicago sativa*, *Hippophae rhamnoides*, *Caragana korshinskii*, *Populus alba* and *Armeniaca vulgaris*, respectively. LM: large macro-aggregates, SM: small macro-aggregates, MI: micro-aggregates, SC: silt/clay.

Linear discriminant analysis effect size (LEfSe) identified that Bacteroidota and Proteobacteria were significantly enriched in AL bulk soils, while Firmicutes, Nitrospirae, and Actinobacteria dominated in NL, MS, and AV, respectively (Figure 6a). Fungal communities showed Mortierellomycota enrichment in AL, Ascomycota in MS, and Basidiomycota in PA (Figure 6c). In soil aggregates, Chloroflexi (bacteria) exhibited peak abundance in small macro-aggregates, whereas Mortierellomycota (fungi) dominated the silt/clay (Figure 6b,d).

### 3.4. Microbial Co-Occurrence Networks

**Figure 7:**
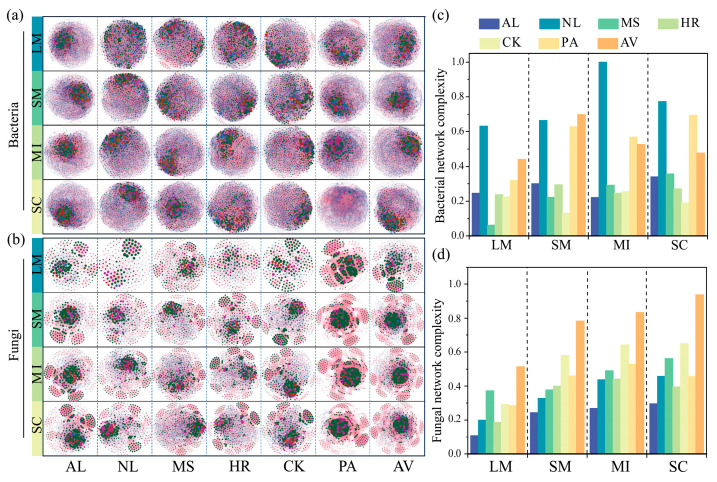
Co-occurrence networks of bacterial (**a**) and fungal (**b**) communities in soil aggregates under different vegetation restoration types. Nodes (colored dots) mean the species that are involved in the networks, the links mean the relationships among the nodes. Red lines represent significant positive (Spearman’s correlation, r > 0.8 and *p* < 0.05) relationships, and blue lines denote (Spearman’s correlation, r < −0.8 and *p* < 0.05) relationships. Bacterial (**c**) and fungal (**d**) network complexity in soil aggregates under different vegetation restoration types. AL: abandoned cropland, NL: natural grassland, MS: *Medicago sativa*, HR: *Hippophae rhamnoides*, CK: *Caragana korshinskii*, PA: *Populus alba*, AV: *Armeniaca vulgaris*, LM: large macro-aggregates, SM: small macro-aggregates, MI: micro-aggregates, SC: silt/clay.

Vegetation restoration altered the microbial co-occurrence patterns in aggregates (Figure 7). The proportions of negative correlations in bacterial and fungal networks ranged from 36.71% to 48.86% and 3.68% to 34.44%, respectively, indicating that microbial interactions were predominantly positive (Figure S3). Compared to AL, NL, PA and AV enhanced the complexity of bacterial networks by increasing the edges/nodes, average degree, average clustering coefficient, and network density, while reducing the average path length; however, HR and CK had the opposite effects (Figure 7c and Figure S3a). All vegetation restoration types increased the complexity of fungal co-occurrence networks relative to AL by elevating the same set of topological indices (Figure 7d and Figure S3b).

### 3.5. Relationships of Soil Abiotic Factors and Microbial Indicators with SMF

**Figure 8:**
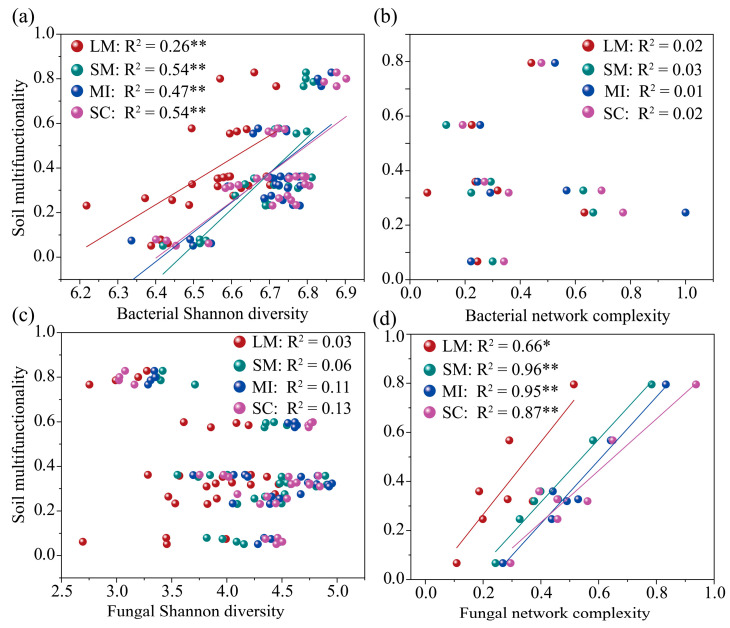
The linear relationships between soil multifunctionality and microbial Shannon diversity (**a**,**c**), and network complexity (**b**,**d**). LM: large macro-aggregates, SM: small macro-aggregates, MI: micro-aggregates, SC: silt/clay. The significance levels are as follows: * *p* < 0.05, ** *p* < 0.01.

**Figure 9:**
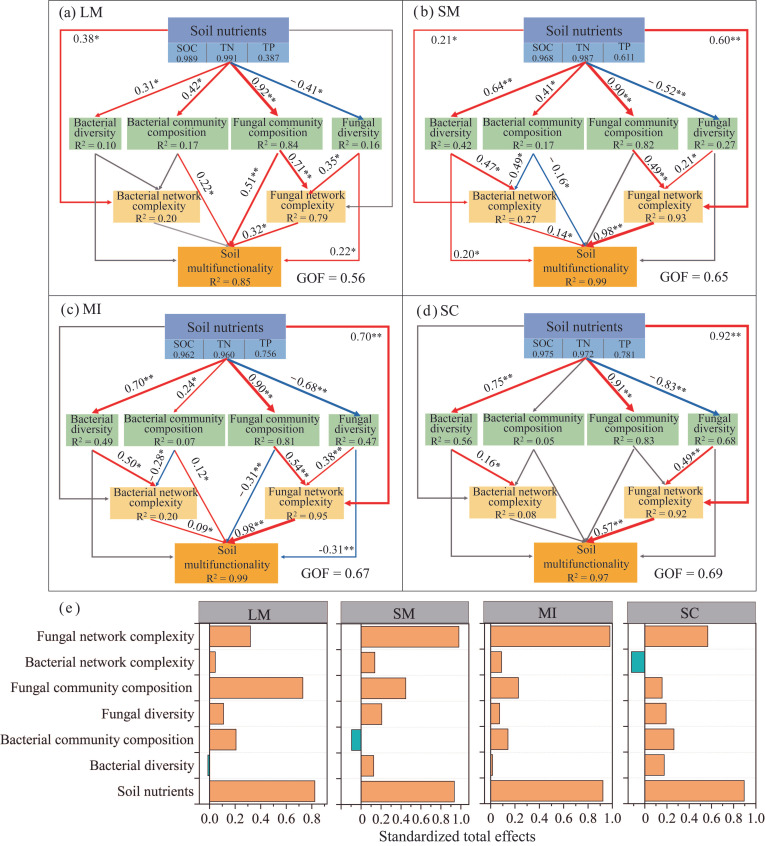
The partial least squares path models (PLS-PM) show direct and indirect pathways influencing soil multifunctionality in (**a**) large macro-aggregates, (**b**) small macro-aggregates, (**c**) micro-aggregates, and (**d**) the silt/clay fraction. Numbers adjacent to arrows denote standardized path coefficients (* *p* < 0.05, ** *p* < 0.01); arrow thickness indicates effect strength; yellow and blue lines represent positive and negative paths, respectively; values in brackets denote the proportion of variance explained for each response variable. Standardized effects from PLS-PM for soil multifunctionality (**e**). Bacterial and fungal communities are represented by the first PCoA axis (PC1). LM: large macro-aggregates, SM: small macro-aggregates, MI: micro-aggregates, SC: silt/clay.

Regression analysis indicated that SMF was positively correlated with bacterial α-diversity and fungal network complexity, with the strongest correlation observed in small macro-aggregates (Figure 8a,d). However, bacterial network complexity and fungal diversity had no significant effect on SMF (Figure 8b,c). The combined effects of soil abiotic factors and microbial indicators accounted for 85%, 99%, 99%, and 97% of the variance in SMF across the LM, SM, MI, and SC fractions, respectively (Figure 9a–d). The fungal community contributed more significantly to SMF than did the bacterial community. Furthermore, fungal network complexity in <2 mm aggregates was the primary microbial predictor of SMF (Figure 9e).

## 4. Discussion

### 4.1. Effects of Vegetation Restoration on Soil Multifunctionality

The ecological restoration of the Loess Plateau has undergone a complex and prolonged process, encompassing both natural restoration dominated by herbaceous species such as *Imperata cylindrica* and artificial restoration measures primarily involving woody vegetation including *Caragana korshinskii*, *Hippophae rhamnoides*, *Prunus armeniaca*, and so on. In this study, vegetation restoration increased soil nutrient concentrations, microbial biomass, and extracellular enzyme activities, but decreased soil pH. Vegetation assimilates atmospheric CO_2_ into organic matter through photosynthesis, which enters the soil as litter, root exudates, and dead roots, thereby directly contributing to the soil organic carbon pool [33,34]. The vegetation restoration process is a synergistic system. Increased soil organic carbon provides energy for nitrogen-fixing and phosphorus-solubilizing microorganisms, thereby enhancing nitrogen and phosphorus accumulation. The improved availability of these nutrients, in turn, stimulates plant growth, further increasing carbon input and sustaining a positive feedback loop [35]. The enhancement of soil microbial biomass during vegetation restoration is a synergistic process driven by three interconnected mechanisms: continuous organic inputs that supply carbon and energy, improved microbial habitats resulting from aggregate formation, and the stimulation of specific microbial taxa via enhanced ecological interactions [19,36]. Plant-derived inputs (litter, fine roots, and exudates) contain abundant structural carbon compounds such as cellulose, hemicellulose, and lignin. In order to utilize these carbon sources, microorganisms secrete a suite of extracellular enzymes, thereby depolymerizing these macromolecules into absorbable sugars [19,37]. Although organic matter inputs introduce nitrogen and phosphorus, these nutrients are largely immobilized in complex organic forms [38,39]. To meet their own nutritional demands and those of plants, microorganisms mineralize these nutrients by secreting N- and P-acquiring enzymes. Our study showed that vegetation restoration stimulated soil acidification, especially in AV, which is consistent with previous reports [40,41]. Generally, returning farmland to forest and grassland stimulated the formation of organic matter, and a high concentration of SOC accelerated the release of organic acids and enhanced the adsorption of H^+^, which led to soil acidification [42]. Furthermore, Guo et al. noted that higher soil aggregation capacities will also reduce soil pH under the action of microorganisms [43]. Therefore, soil acidification represents a product of the integrated soil–plant–microbe interactions during vegetation restoration.

Vegetation restoration significantly enhanced soil C, N, and P cycling and multifunctionality relative to AL, particularly in the AV. SMF integrates many different ecosystem functions, including nutrient provisioning, nutrient cycling, and microbial growth efficiency [7,44]. We found that vegetation restoration increased soil nutrient concentrations, microbial biomass and extracellular enzyme activity. These findings demonstrate that vegetation restoration establishes positive feedback on SMF through increased nutrient availability, enhanced microbial biomass, and stimulated enzyme activities [35,45]. Vegetation restoration may intensify water uptake and transpiration by plant roots, which reduces deep soil moisture in semi-arid or arid regions [46,47]. This moisture depletion can suppress microbial activity and slow the decomposition rates of soil organic carbon and nitrogen, thereby exerting a negative impact on SMF. Furthermore, vegetation restoration can lead to soil nutrient imbalances and limitations, such as increased carbon but decreased phosphorus availability, thereby disrupting the balance of SMF [48]. Future research should employ multi-scale and integrated approaches to elucidate the intrinsic mechanisms through which vegetation restoration influences SMF.

### 4.2. Effects of Vegetation Restoration on Soil Microbial Communities

Our mixed-effects model showed that the contribution of vegetation types to the change in microbial diversity was higher than that of aggregate fractions. Vegetation restoration serves as the primary driver of soil ecosystem transformation, modifying microbial activity and community structure through multiple direct and indirect pathways [7,49]. Although soil aggregates provide habitats for microorganisms, their formation and stability are strongly influenced by the vegetation inputs and microbial activities [19]. We found that vegetation restoration enriched bacterial Shannon diversity in bulk soil and aggregates, indicating that vegetation restoration attracted more specific bacterial groups through resource input [18]. However, the fungal diversity of aggregates showed that MS and CK were significantly higher than AL, while AV and HR were significantly lower than AL. This is likely due to the greater canopy coverage in AV and HR, which limited light availability for understory plants and thus reduced their diversity [50]. The reduction in vegetation diversity reduces soil fungal diversity through limited inputs of organic matter [51]. Wang et al. also found that different plant species create complementary belowground niches by providing specific host plants for arbuscular mycorrhizal fungi and pathogenic fungi, along with varied resources for saprotrophic fungi, thus fostering multidimensional interactions with plant diversity [52].

Our study reported that the bacterial Shannon index was higher in <2 mm aggregates than in large macro-aggregates, while the fungal Shannon index was higher in micro-aggregates than in both large and small macro-aggregates. Micro-aggregates and small macro-aggregates possess smaller mean pore sizes than large macro-aggregates, thereby effectively protecting microorganisms from predation by larger predators (e.g., protozoa and nematodes) and thus providing a secure habitat for bacteria and fungi [13,53]. This protective capacity also further enhances resilience to external physical disturbances and environmental fluctuations, such as dry-wet alternation [54]. Furthermore, the relatively abundant organic matter in micro-aggregates stimulates microbial enrichment, whereas large macro-aggregates are usually dominated by fresh organic materials (e.g., litter and roots), which triggers rapid proliferation of r-strategists and intense competition, thereby reducing microbial diversity [55]. In general, resource heterogeneity within microaggregates supports a highly diverse microbial community by providing a variety of ecological niches.

The bacterial taxa Bacteroidota and Proteobacteria and fungal taxa Mortierellomycota were significantly enriched in AL soils, while Actinobacteria and Basidiomycota dominated in AV and PA soils, respectively. Moreover, Chloroflexi (bacteria) exhibited peak abundance in small macro-aggregates, whereas Mortierellomycota (fungi) dominated in silt/clay, supporting our hypothesis that different vegetation restoration strategies and aggregate fractions significantly changed microbial communities. These findings may be predominantly ascribed to disparities in vegetation composition and soil physicochemical characteristics [56]. Muneer et al. found that the relative abundance of Bacteroidota and Chloroflexi was significantly positively correlated with soil pH in orchard red soil [57]. Interestingly, the fungal taxon Mortierellomycota was significantly enriched in nutrient-deficient and strongly alkaline environments, such as in AL and silt/clay, suggesting that Mortierellomycota is an oligotrophic fungus. However, Basidiomycota predominantly colonize resource-rich environments, demonstrating specialized capacities for lignocellulose degradation and utilization [58,59]. Previous studies have consistently reported significant enrichment of Actinobacteria in the calcareous soils of the Loess Plateau [60,61,62]. In our study, Actinobacteria were significantly enriched in AV compared to other vegetation types, which may be attributed to the richer nutrient conditions in AV. Liao et al. also have demonstrated that grassland restoration enhances the relative abundance of Actinobacteria by increasing SOC content and enzyme activities [61]. These findings collectively indicate that Actinobacteria represent a copiotrophic bacterial phylum characterized by their preferential colonization of resource-rich environments.

Microbial networks reflect the potential relationships of competition or cooperation between species [63]. The bacterial-fungal co-occurrence networks across all sampling sites were predominantly characterized by positive correlations in this study, suggesting prevalent mutualistic interactions within these soil microbial communities. Previous studies have demonstrated that microorganisms employ cooperative strategies to mitigate environmental stresses (e.g., low temperature and drought), particularly in cold-arid regions where such interactions are critical for maintaining metabolic functionality under extreme abiotic constraints [64,65]. Interestingly, the edges/nodes, average degree, and proportion of negative correlations in fungal co-occurrence networks reached their maximum values in silt/clay, likely attributable to intensified inter-taxa competition driven by nutrient scarcity within the oligotrophic microenvironment of silt/clay [56].

Soil microbial community complexity plays a pivotal role in sustaining ecosystem functioning and services through its regulation of biogeochemical cycles, resilience to environmental perturbations, and mediation of plant–soil feedback mechanisms [27,66]. Our findings demonstrate that vegetation restoration increased the complexity of fungal networks in aggregates, especially AV. Vegetation restoration significantly improves soil quality and provides microorganisms with more abundant nutrient sources and expanded habitats, thereby promoting microbial growth and metabolic activities, which consequently enhances the complexity of microbial networks [7,45]. Furthermore, we found that oligotrophic microorganisms (such as Mortierellomycota) were significantly enriched in AL, indicating that vegetation restoration shifts microbial communities from oligotrophic to copiotrophic strategies, thereby promoting more diversified interactions among distinct microbial taxa [61,67]. However, HR and CK decreased the complexity of bacterial co-occurrence networks relative to AL in this study. This finding is contrary to our expectation, suggesting that a decrease in microbial network complexity cannot be simply equated with ecosystem degradation, which may be attributed to the weaker competitive interactions among bacteria in HR and CK, which consequently reduced the complexity of the bacterial network [68]. Therefore, changes in soil microbial network complexity are highly contingent on the specific ecosystem, vegetation restoration approaches, and the resultant environmental conditions.

### 4.3. Effects of Abiotic and Biotic Drivers on Soil Multifunctionality

Soil bacterial diversity was a stronger predictor of SMF than fungal diversity, which was supported by [8,69]. Soil bacteria possess a wider range of metabolic pathways and functional diversity, which participate in virtually all known soil biogeochemical processes and can simultaneously perform multiple ecological functions [70]. Furthermore, bacterial communities respond to environmental changes more rapidly and sensitively, with their diversity and composition being more readily altered by variations in environmental conditions such as precipitation, soil pH, and nutrient availability [71]. Consequently, following ecological restoration or environmental disturbance, bacterial communities can rapidly adapt and subsequently restore their ecological functions. Both bacterial and fungal community composition contributed positively to SMF, which is potentially mediated by the vegetation restoration-induced shift in microbial community composition from oligotrophic to copiotrophic groups, thereby stimulating organic matter decomposition and consequently enhancing SMF [61,72]. Fungal communities exhibit strong phylogenetic conservation of functional traits (e.g., lignin/cellulose degradation, mycorrhizal type), rendering them functionally unique and less replaceable [73]. Although bacterial communities exhibit high phylogenetic diversity, they possess substantial functional redundancy, thus weakening the direct impact on SMF [74]. Vegetation restoration stimulated the metabolic process of microbial communities, resulting in more microbial residues entering the soil and ultimately contributing to soil organic matter [18]. Fungal residues, as a primary contributor to soil organic carbon, largely promote the accumulation of soil nutrients, thereby exerting a positive influence on SMF [75]. In summary, the critical contribution of stable fungal community structures to ecosystem functioning must be considered in vegetation restoration strategies for arid and semi-arid regions.

Microbial network complexity is recognized as an essential contributor to maintaining SMF [12,27,76]. However, relatively few studies have linked microbial community characteristics across different aggregate sizes with SMF and have thoroughly investigated the differential mechanisms of microbial-mediated SMF changes at the aggregate scale, especially during vegetation restoration on the Loess Plateau. Our study revealed that fungal network complexity served as the primary microbial predictor of SMF variation in <2 mm aggregates, whereas in large macro-aggregates, it was fungal community composition, which contradicted our third hypothesis. The fine pores of <2 mm aggregates provide a stable and secure environment for fungal hyphae by shielding them from larger predators and buffering physical disturbances, thereby facilitating the establishment of complex networks [13]. Concurrently, fungi can promote the formation of stable aggregates through physical and biochemical mechanisms (e.g., hyphal entanglement and exudation of adhesive compounds), thereby effectively sequestering nutrients within the soil matrix [77]. In this study, the <2 mm aggregates exhibited higher fungal diversity and negative correlations in the edge ratio, which also supports this conclusion.

This study explored the effects of vegetation restoration on soil factors, microbial characteristics, and ecological regulation pathways of SMF in the Loess Plateau. Therefore, given the variations in belowground multitrophic communities across different soil aggregates [78], future studies should adopt integrated multi-scale and multi-factor approaches to investigate the micro-ecological mechanisms that sustain SMF, thereby establishing a theoretical framework for developing optimal vegetation restoration strategies in this region.

## 5. Conclusions

In summary, this study investigated the effects of different vegetation restoration strategies on microbial community characteristics, network complexity, and SMF at the aggregate scale on the Loess Plateau and further explored the key biotic factor driving SMF variations. Our findings demonstrated that vegetation restoration reshaped microbial community structure, enhancing bacterial diversity, fungal network complexity, and SMF, particularly in AV. Fungal diversity and bacterial network complexity exhibited asynchronous dynamics across different-sized aggregates during vegetation restoration. Fungal community composition was the key predictor of SMF in large macro-aggregates, whereas fungal network complexity played the dominant role in <2 mm aggregates. Soil nutrient concentration plays an important role in driving SMF variation by shaping microbial community structure. This study advances our understanding of how soil microbial communities sustain ecosystem functioning in the Loess Plateau, thereby offering scientific support for designing effective restoration strategies.

## Data Availability

The original contributions presented in this study are included in the article and Supplementary Materials. Further inquiries can be directed to the corresponding author.

## Supplementary Materials

The following supporting information can be downloaded at: https://www.mdpi.com/article/10.3390/microorganisms14010161/s1, Figure S1: Soil biogeochemical properties under different vegetation restoration types; Figure S2: Soil pH and nutrient concentrations in aggregates under different vegetation restoration types; Figure S3: Co-occurrence network properties of bacteria (a) and fungi (b) in different vegetation types under different aggregate particle sizes; Table S1: Methods for determination of soil physicochemical and biological properties; Table S2: Basic situation of vegetation types of study area.
