# Supplementary material for: Soil Aggregate Fungal Network Complexity Drives Soil Multifunctionality During Vegetation Restoration
Source: Microorganisms. 2026 Jan 11;14(1):161. doi: 10.3390/microorganisms14010161 (PMC12843732; doi:10.3390/microorganisms14010161)
Supplement: Supplementary file 1 [file microorganisms-14-00161-s001.zip › microorganisms-4036165-supplementary.pdf]

Table S1 Basic situation of vegetation types of study area

Vegetation types	Geographical location	Elevation (m)	Aspect	Main species
AL	104.7609°E 35.4911°N	2040.34	Sunny slope	*Agropyron cristatum, Imperata cylindrica, Artemisia frigida Willd*
NL	104.7601°E 35.4900°N	2064.14	Sunny slope	*Imperata cylindrica, Stipa capillata, Agropyron cristatum, Artemisia frigida Willd*
MS	104.7627°E 35.4926°N	2029.03	Sunny slope	*Medicago sativa, Agropyron cristatum, Thymus mongolicus, Peganum harmala*
HR	104.7594°E 35.4903°N	2062.24	Sunny slope	*Hippophae rhamnoides, Agropyron cristatum, Thymus mongolicus, Thermopsis lanceolata, Stipa capillata*
CK	104.7605°E 35.4937°N	2057.67	Sunny slope	*Caragana korshinskii, Peganum harmala, Eleusine indica, Agropyron cristatum, Ceratoides latens*
PA	104.7605°E 35.4916°N	2037.10	Sunny slope	*Populus alba, Thermopsis lanceolata, Agropyron cristatum, Imperata cylindrica, Gueldenstaedtia verna*
AV	104.7597°E 35.4931°N	2067.00	Sunny slope	*Armeniaca vulgaris, Agropyron cristatum, Artemisia frigida Willd, Tripogon chinensis, Peganum harmala*

AL: abandoned cropland, NL: natural grassland, MS: *Medicago sativa*, HR: *Hippophae rhamnoides*, CK: *Caragana korshinskii*, PA: *Populus alba*, AV: *Armeniaca vulgaris.*

Table S2 Methods for determination of soil physicochemical and biological properties

Soil properties	Methods	Soil properties	Methods
pH (2.5:1)	It was determined by a digital pH meter (Sartorius PB-10, Goettingen, Germany) in a suspension with a soil-to-water ratio of 1:2.5.	Easily oxidizable organic carbon (EOC)	It was extracted with 333 mmol/L KMnO4 and then assayed by a spectrophotometer (METASH V-5800, Shanghai, China).
Soil organic carbon (SOC)	Wet oxidation with KCr_2O_7 + H_2SO_4 and titration with $FeSO_4$ were used to determine it.	Microbial biomass carbon (MBC)	After chloroform fumigation extraction, a CN 802 Carbon Nitrogen Elemental Analyzer (VELP Scientifica, Usmate, Italy) was used to determine it.
Total nitrogen (TN)	It was determined by the semi-micro Kjeldahl method.	Microbial biomass nitrogen (MBN)	After chloroform fumigation extraction, a CN 802 Carbon Nitrogen Elemental Analyzer (VELP Scientifica, Usmate, Italy) was used to determine it.
Total phosphorus (TP)	It was determined by the ammonium molybdate method after $HClO_4$-H_2SO_4 digestion.	Microbial biomass phosphorus (MBP)	After chloroform fumigation extraction, the ammonium molybdate ascorbic acid technique was used to determine it.
Available phosphorus (AP)	Following extraction with 0.5 M $NaHCO_3$, the ammonium molybdate method was used to determine it.	β-glucosidase (BG)	It was determined by a SpectraMax 190 microplate reader (Molecular Devices, America), and the reaction substrate is p-Nitrophenyl β-D-Glucopyranoside.
Nitrate nitrogen (NO_3^--N)	It was extracted with 2 M KCl (soil:solution = 1:5 (w/v)), and the extracts were analyzed by a continuous flow analyzer (Auto-Analyzer AA3, Germany).	N-acetylglucosaminidase (NAG)	It was determined by a SpectraMax 190 microplate reader (Molecular Devices, America), and the reaction substrate is p-Nitrobenzene β-N-Acetylglucosamine.
Nmmonium nitrogen (NH_4^+-N)	It was extracted with 2 M KCl (soil:solution = 1:5 (w/v)), and the extracts were analyzed by a continuous flow analyzer (Auto-Analyzer AA3, Germany).	Leucine aminopeptidase (LAP)	It was determined by a SpectraMax 190 microplate reader (Molecular Devices, America), and the reaction substrate is L-leucine p-nitroaniline.
Dissolved organic carbon (DOC)	It was extracted with ultrapure water (soil:solution = 1:5 (w/v)), and the extracts were analyzed with a CN 802 Carbon Nitrogen Elemental Analyzer (VELP Scientifica, Usmate, Italy).	Alkaline phosphatase (ALP)	It was determined by a SpectraMax 190 microplate reader (Molecular Devices, America), and the reaction substrate is disodium phenyl phosphate.

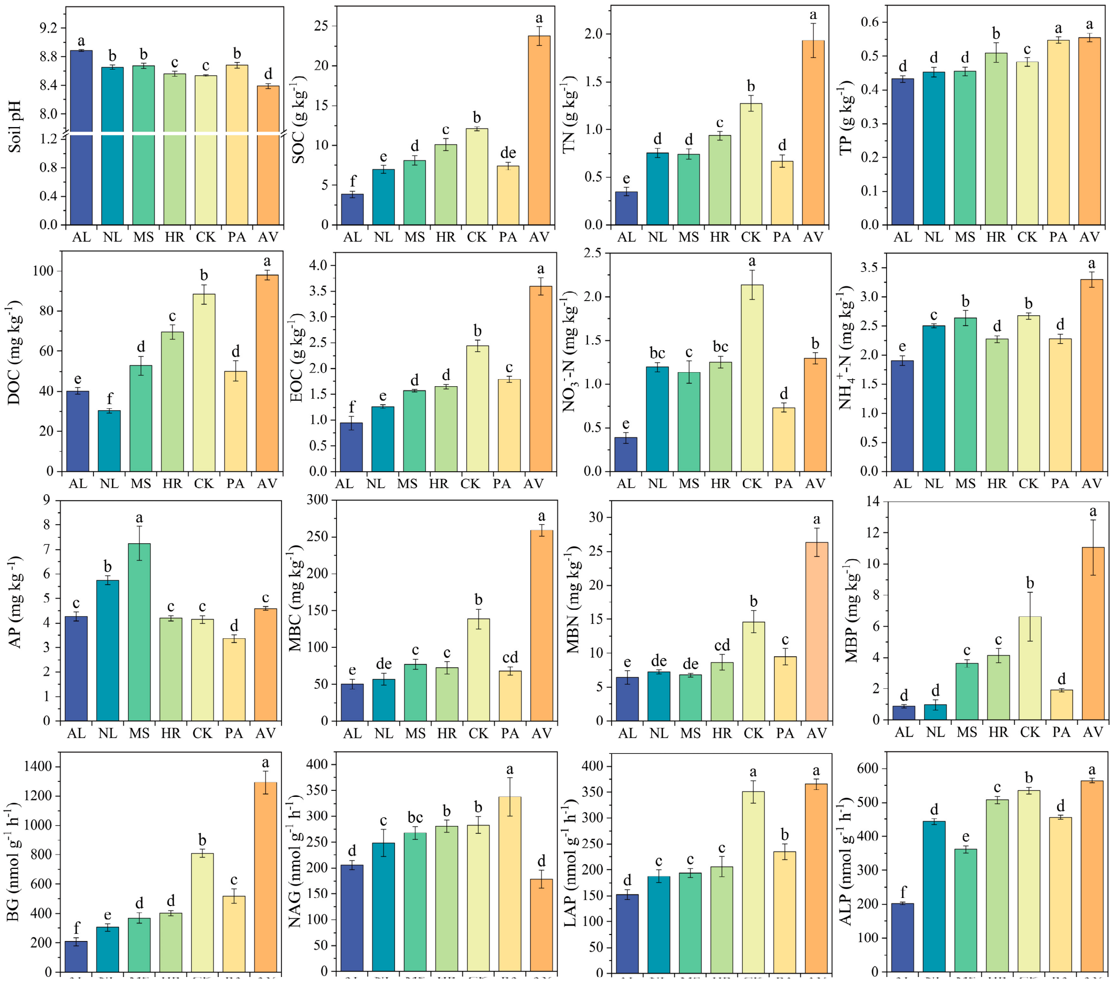

Figure S1. Soil biogeochemical properties under different vegetation restoration types. Different lowercase letters indicate significant differences among vegetation types according to the least significant difference (LSD) test ($p < 0.05$). AL, NL, MS, HR, CK, PA and AV represent abandoned cropland, grassland, *Medicago sativa*, *Hippophae rhamnoides*, *Caragana korshinskii*, *Populus alba* and *Armeniaca vulgaris*, respectively. SOC, Soil organic carbon; TN, total nitrogen; TP, total phosphorus; DOC, dissolved organic carbon; EOC, easily oxidizable organic carbon; NO_3^--N, nitrate nitrogen; NH_4^+-N, ammonium nitrogen; AP, available phosphorus; MBC, microbial biomass carbon; MBN, microbial biomass nitrogen; MBP, microbial biomass phosphorus; BG, *β*-glucosidase; NAG, *N*-acetylglucosaminidase; LAP, leucine aminopeptidase; ALP, alkaline phosphatase.

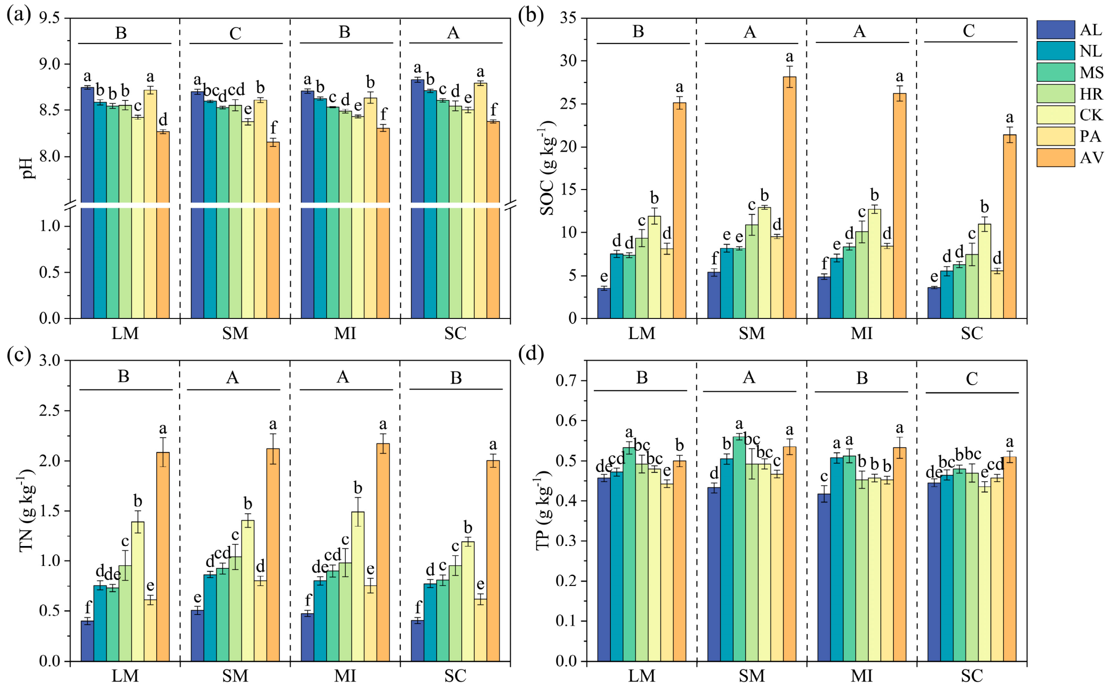

Figure S2. Soil pH and nutrient concentrations in aggregates under different vegetation restoration types. Different lowercase letters indicate significant differences among vegetation restoration types within the same aggregate size, and different uppercase letters indicate significant differences among aggregate sizes according to the least significant difference (LSD) test ($p < 0.05$). AL: abandoned cropland, NL: natural grassland, MS: *Medicago sativa*, HR: *Hippophae rhamnoides*, CK: *Caragana korshinskii*, PA: *Populus alba*, AV: *Armeniaca vulgaris*, LM: large macro-aggregates, SM: small macro-aggregates, MI: micro-aggregates, SC: silt/clay.

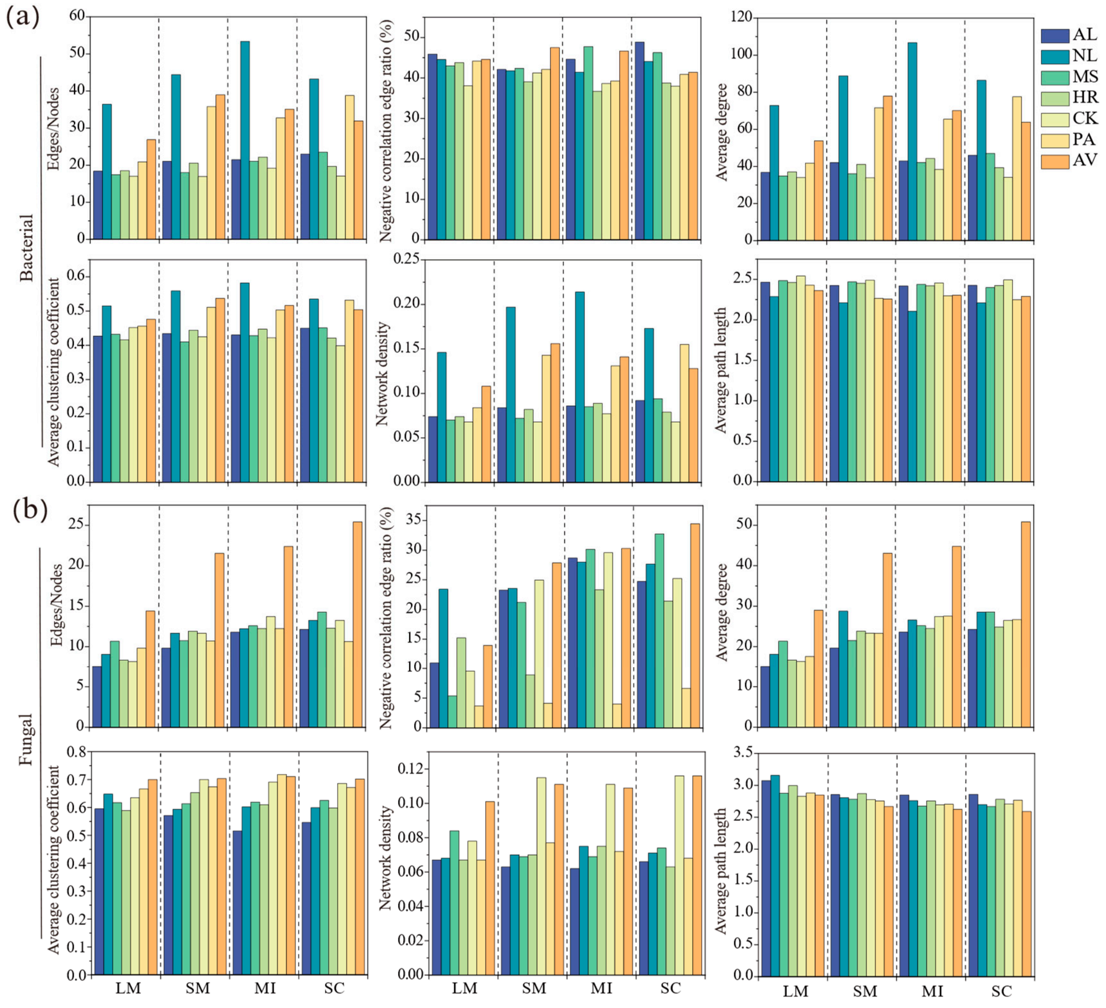

Figure S3. Co-occurrence network properties of bacteria (a) and fungi (b) in different vegetation types under different aggregate particle sizes. AL, NL, MS, HR, CK, PA and AV represent abandoned cropland, grassland, *Medicago sativa*, *Hippophae rhamnoides*, *Caragana korshinskii*, *Populus alba* and *Armeniaca vulgaris*, respectively. LM: large macro-aggregates, SM: small macro-aggregates, MI: micro-aggregates, SC: silt/clay.
